# Evaluation of an open forecasting challenge to assess skill of West Nile virus neuroinvasive disease prediction

**DOI:** 10.1186/s13071-022-05630-y

**Published:** 2023-01-12

**Authors:** Karen M. Holcomb, Sarabeth Mathis, J. Erin Staples, Marc Fischer, Christopher M. Barker, Charles B. Beard, Randall J. Nett, Alexander C. Keyel, Matteo Marcantonio, Marissa L. Childs, Morgan E. Gorris, Ilia Rochlin, Marco Hamins-Puértolas, Evan L. Ray, Johnny A. Uelmen, Nicholas DeFelice, Andrew S. Freedman, Brandon D. Hollingsworth, Praachi Das, Dave Osthus, John M. Humphreys, Nicole Nova, Erin A. Mordecai, Lee W. Cohnstaedt, Devin Kirk, Laura D. Kramer, Mallory J. Harris, Morgan P. Kain, Emily M. X. Reed, Michael A. Johansson

**Affiliations:** 1 Global Systems Laboratory, National Atmospheric and Oceanic Administration, Boulder, CO USA; 2 Division of Vector-Borne Diseases, Centers for Disease Control and Prevention, Fort Collins, CO USA; 3 Department of Pathology, Microbiology, and Immunology, School of Veterinary Medicine, University of California, Davis, CA USA; 4 Division of Infectious Diseases, Wadsworth Center, New York State Department of Health, Albany, NY USA; 5 Department of Atmospheric and Environmental Sciences, University at Albany, Albany, NY USA; 6 Evolutionary Ecology and Genetics Group, Earth & Life Institute-UCLouvain, Louvain-La-Neuve, Belgium; 7 Emmett Interdisciplinary Program in Environment and Resources, Stanford University, Stanford, CA USA; 8 Information Systems and Modeling, Los Alamos National Laboratory, Los Alamos, NM USA; 9 Center for Vector Biology, Rutgers University, New Brunswick, NJ USA; 10 Department of Medicine, University of California, San Francisco, CA USA; 11 Department of Mathematics and Statistics, Mount Holyoke College, South Hadley, MA USA; 12 Department of Pathobiology, University of Illinois at Urbana-Champaign, Urbana, IL USA; 13 Department of Environmental Medicine and Public Health, Icahn School of Medicine at Mount Sinai, New York, NY USA; 14 Department of Global Health, Icahn School of Medicine at Mount Sinai, New York, NY USA; 15 Biomathematics Graduate Program, North Carolina State University, Raleigh, NC USA; 16 Department of Entomology, Cornell University, Ithaca, NY USA; 17 Statistical Sciences Group, Los Alamos National Laboratory, Los Alamos, NM USA; 18 Agricultural Research Service, United States Department of Agriculture, Sidney, MT USA; 19 Department of Biology, Stanford University, Stanford, CA USA; 20 National Bio- and Agro-Defense Facility, Agricultural Research Service, United States Department of Agriculture, Manhattan, KS USA; 21 Invasive Species Working Group, Global Change Center, Fralin Life Sciences Institute, Virginia Tech, Blacksburg, NC USA; 22 Division of Vector-Borne Diseases, Centers for Disease Control and Prevention, San Juan, PR USA

**Keywords:** Calibration, Discriminatory power, Forecasting, Logarithmic score, Multi-model assessment, West Nile virus, West Nile neuroinvasive disease, United States

## Abstract

**Background:**

West Nile virus (WNV) is the leading cause of mosquito-borne illness in the continental USA. WNV occurrence has high spatiotemporal variation, and current approaches to targeted control of the virus are limited, making forecasting a public health priority. However, little research has been done to compare strengths and weaknesses of WNV disease forecasting approaches on the national scale. We used forecasts submitted to the 2020 WNV Forecasting Challenge, an open challenge organized by the Centers for Disease Control and Prevention, to assess the status of WNV neuroinvasive disease (WNND) prediction and identify avenues for improvement.

**Methods:**

We performed a multi-model comparative assessment of probabilistic forecasts submitted by 15 teams for annual WNND cases in US counties for 2020 and assessed forecast accuracy, calibration, and discriminatory power. In the evaluation, we included forecasts produced by comparison models of varying complexity as benchmarks of forecast performance. We also used regression analysis to identify modeling approaches and contextual factors that were associated with forecast skill.

**Results:**

Simple models based on historical WNND cases generally scored better than more complex models and combined higher discriminatory power with better calibration of uncertainty. Forecast skill improved across updated forecast submissions submitted during the 2020 season. Among models using additional data, inclusion of climate or human demographic data was associated with higher skill, while inclusion of mosquito or land use data was associated with lower skill. We also identified population size, extreme minimum winter temperature, and interannual variation in WNND cases as county-level characteristics associated with variation in forecast skill.

**Conclusions:**

Historical WNND cases were strong predictors of future cases with minimal increase in skill achieved by models that included other factors. Although opportunities might exist to specifically improve predictions for areas with large populations and low or high winter temperatures, areas with high case-count variability are intrinsically more difficult to predict. Also, the prediction of outbreaks, which are outliers relative to typical case numbers, remains difficult. Further improvements to prediction could be obtained with improved calibration of forecast uncertainty and access to real-time data streams (e.g. current weather and preliminary human cases).

**Graphical Abstract:**

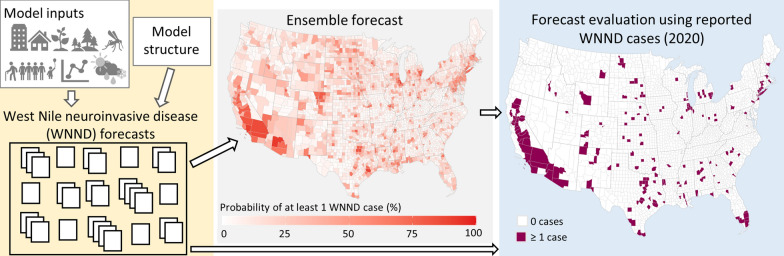

**Supplementary Information:**

The online version contains supplementary material available at 10.1186/s13071-022-05630-y.

## Background

West Nile virus (WNV; *Flaviviridae, Flavivirus*) is the leading cause of mosquito-borne illness in the continental USA [1]. Symptomatic infections typically present as a febrile illness (approximately 20% of all infections). However, < 1% of all infections result in West Nile neuroinvasive disease (WNND) with manifestations including meningitis, encephalitis, or acute flaccid paralysis [2]. WNV was first detected in the US in 1999 [3] and by 2004, had spread across the contiguous US and up the Pacific coast [4]. From 1999 to 2020, the Centers for Disease Control and Prevention (CDC) reported a total of 26,683 non-neuroinvasive WNV disease cases and 25,849 WNND cases, resulting in 2456 deaths [5]. Since WNV became endemic (2005–2020), a median of 409 (range 167–693; 5–22%) of the 3108 counties in the contiguous US report WNND cases each year. WNV exhibits marked seasonality with most cases reported between July and October nationwide [5]. Even in counties that regularly report WNND cases, the number and location of WNND cases vary. For example, reported WNND cases per county can range from singles to a few dozen or 50 with 239 cases reported in the largest outbreak during this time [6]. Large spatial and temporal heterogeneity in annual WNND cases makes accurate prediction of incidence both challenging and potentially valuable to guide prevention and control efforts.

The ecology of WNV is complex and spatially variable across the US. The virus is maintained in an enzootic cycle between birds (predominantly passerines) and *Culex* mosquitoes [7–9], but can cause disease in horses and humans, which are dead-end hosts [10]. The vectors for WNV vary geographically [9]. In the east-central region (Northeast, mid-Atlantic, and central US), *Cx. pipiens* and *Cx. restuans* have been incriminated as the primary vectors with *Cx. salinarius* also playing an important role in maintenance and zoonotic transmission in coastal areas. In the southeast, *Cx. quinquefasciatus* has been implicated as the primary vector with *Cx. salinarius* and *Cx. nigripalpus* also capable of causing human disease. In western North America, *Cx. tarsalis* is largely responsible for zoonotic transmission, especially in more rural areas, while *Cx. pipiens* serves as the enzootic vector in urban areas in the more northern parts of the western US (northern Great Plains, Rocky Mountains, and Pacific Northwest). In urban areas of the southwestern US, *Cx. quinquefasciatus* can act as the dominant zoonotic vector. Other *Culex* mosquito species can have a secondary or localized importance in this region.

Meteorological factors like temperature and precipitation have a large impact on the transmission of WNV. Temperature influences mosquito survival and potential WNV transmission rates [11]. As temperatures warm, mosquito development and biting rates accelerate [11, 12]. Additionally, with increasing temperature, the extrinsic incubation period for WNV decreases as viral replication rates increase [13–16]. Thus, with increasing temperature above the thermal minimum for mosquito survival and WNV replication [15, 17], viral transmission and risk of zoonotic transmission increase. However, there is a thermal optimum (23.9–25.2 °C [18]) above which transmission generally decreases because of negative impacts on mosquito survival and other traits. Variation in the interaction of climatic and landscape factors contributes to seasonal dynamics and spatial variation in the effect of temperature [9, 19]. Increased precipitation generally increases the quantity of available larval habitat [20–22], but intense precipitation events can wash out immature mosquitoes from larval habitat such as catch basins [23]. The impact of precipitation varies broadly across the US with a positive association between increased precipitation and above average human cases in the western US, but a negative association in the eastern US. This difference is potentially due to difference in the mosquito species, their preferred egg-laying habitats, and other environmental factors present in each area [9, 19, 22]; in the West, increased precipitation likely leads to increased *Cx. tarsalis* larval habitats, while in the East, increased precipitation may wash out *Cx. pipiens* larval habitats. Also, drought has been associated with WNV amplification and increased human cases, partially due to aggregation of hosts and vectors at dwindling water sources [24, 25].

Statistical and mechanistic models have been developed to predict geographic or temporal dynamics of WNV transmission [26, 27]. These models included some subset of the following grouping of variables: historical human cases, veterinary cases, climate, hydrology, human demographics, land use, viral genetics, mosquito surveillance, sentinel surveillance, and avian population dynamics. Models generally produce estimates on a single spatial and temporal scale aimed at guiding public health decisions or elucidating factors that enable increased transmission. Models developed for prediction in one location often fail to perform well if applied to a different location because of variation in factors like ecology, primary mosquito species, and human behavior as well as availability of predictor data, such as mosquito surveillance data [28]. Out-of-sample validation is often used to assess model performance, but no multi-model comparative assessment has been performed to assess the strengths and weaknesses of predictive WNV modeling at the local or national scale.

To systematically evaluate WNND prediction across the continental US, the CDC Epidemic Predictive Initiative and the Council for State and Territorial Epidemiologists launched an open West Nile virus Forecasting Challenge in 2020. The primary objective of the Challenge was to predict the total number of WNND cases for each county in the contiguous US that would be reported to the national surveillance system for arboviral diseases, ArboNET, during the 2020 calendar year. In our evaluation of the Challenge, we (i) assessed whether some models had better predictive performance than others, (ii) identified modeling approaches associated with better prediction, and (iii) evaluated contextual factors of the counties (e.g. environmental, climatic, and historical WNV patterns) associated with variation in forecast skill.

## Methods

### Team participation

An announcement recruiting team participation in the 2020 WNV Forecasting Challenge was circulated widely by the CDC Epidemic Prediction Initiative through emails and postings on webpages starting in March 2020. Teams using any modeling approach were encouraged to participate.

Participating teams signed a data use agreement and were provided with annual WNND case counts by county for the contiguous US and Washington DC during 2000–2018 from ArboNET, the national arboviral diseases surveillance system administered by the CDC. Provisional 2019 case data were provided to participants in early May 2020. Participants were allowed to use any other data source, like climate, weather, land use, mosquito surveillance, and human demographics, at whatever spatial and temporal scaled they deemed appropriate to develop their modeling approach. See Additional file 1: Text S1 for details on modeling methodologies and datasets used by each team.

### Forecasting target

Teams predicted the total number of probable and confirmed WNND cases that would be reported to ArboNET for all counties (*n* = 3108) in the contiguous US and Washington DC during 2020. WNND cases were chosen as the outcome because the severe manifestations of the disease are more likely to be consistently recognized and reported compared with less severe, non-neuroinvasive WNV disease cases [29].

For each county, a forecast included both a point estimate and a binned probability distribution. The point estimate denoted the most likely number of cases. Fifteen bins were chosen to cover the range of cases from 0 to > 200, reflecting a typical range of observed cases across counties, with finer resolution for smaller numbers of expected cases given the relatively few cases reported in the majority of counties (i.e. bins for 0, 1–5, 6–10, …, 46–50, 51–100, 101–150, 151–200, > 200 cases). These bins provide meaningful information for location-specific public health action given that, on average, 0.38 WNND cases per county are reported each year (on average, 88% of counties report zero cases, 11.5% report 1–10 cases, and 0.4% report 11–50 cases with yearly county maximums ranging from 18–239 cases, 2005–2020) [6]. Teams assigned a probability between 0 and 1 to each bin, with a total probability equal to 1.0 across all bins per county.

### Forecasts

The initial forecast due date was April 30, 2020, with submission to an online system (https://predict.cdc.gov). Additional, optional, updated submissions could be submitted by the following deadlines: May 31, June 30, and July 31, 2020. Further details are available through the project’s GitHub repository (https://github.com/cdcepi/WNV-forecast-project-2020).

Concurrently, we developed four additional models of varying complexity and use of historical case data for comparison with the team forecasts: a naïve model, an always-absent model, a negative binomial model, and an ensemble model. The naïve model used no historical data and assigned equal probability to each of the bins (i.e. 1/15 probability). The always-absent model also ignored historical data and represented a universal expectation of zero cases by assigning a probability of 1.0 to the zero-case bin and zero probability to all other bins for each county. We included this model given the relatively small percent of counties in the US that report WNND cases each year. The negative binomial model was built to reflect a parsimonious probabilistic prediction relying exclusively on local historical data, a “same-as-before” baseline model. For each county, we fitted a negative binomial distribution to historical WNND cases and extracted probabilities for each bin from the cumulative distribution function. The initial version of this forecast (April submission) used 2000–2018 case counts, while the May submission also incorporated the provisional 2019 data reported as of May 2020. Finally, we created a mean consensus ensemble using all team-submitted forecasts and the negative binomial forecast by averaging the probabilities assigned in each bin for all forecasts at each location and submission deadline. For forecasts that were not updated at a particular submission deadline, we used the last available forecast for each update of the ensemble. Using the final version of the ensemble, we used Shannon entropy [30] to assess the spread of probability across the binned case counts (uncertainty) in the ensemble model forecast.

We developed two additional models retrospectively as alternative baseline models: a first-order autoregressive model (i.e. AR(1)) and a first-order autoregressive model with a single climate variable as an exogenous covariate (AR(1) Climate). For both models, we fitted log-transformed annual WNND case counts (2005–2019; ln(cases + 1)) using the *arima* function in the stats package in R (version 4.1.2; [31]). For the AR(1) Climate model, we considered seasonal aggregations of climate conditions (i.e. average temperature, mean minimum temperature, or total precipitation), using Parameter-elevation Regressions on Independent Slopes Model (PRISM) data [32] aggregated to county. We defined seasons as 3-month periods for winter (December–February), spring (March–May), summer (June–August), and fall (September–November). To predict annual WNND case numbers, we considered including climate data from the previous winter to the concurrent year’s spring to capture any lagged climate-induced impacts on transmission during the previous year (e.g. considering seasonal climate data from December 2018–May 2020 to predict 2020 WNND cases). See Additional file 1: Text S1 for more details on the development of the autoregressive modeling framework.

### Evaluation

As announced before the Challenge, we evaluated all forecasts using the logarithmic score, a proper scoring rule based on the probabilities assigned in each forecast in relation to the eventual observed case counts [33, 34]. The score for each team was the average logarithm of the probability assigned to the observed outcome bin, the bin containing the reported number of WNND cases for 2020, per county. To avoid logarithmic scores of negative infinity for forecasts which assigned zero probability to the observed outcome, we truncated binned predictions to have a minimum logarithmic score of − 10. We compared mean logarithmic scores with ANOVA followed by Tukey post hoc multiple comparisons to identify significant differences between forecast scores. We compared the forecasts for the final versions of team forecasts and comparison models and between the initial and final versions of all forecasts.

We assessed probabilistic calibration by plotting forecasted probabilities versus observed frequencies for forecasts with each summarized in the following upper-bound inclusive probability bins: 0.0, 0.0–0.1, 0.1–0.2, …, 0.9–1.0. Note that these bins are the probabilities assigned to case number bins, not the cases number bins themselves. We then calculated a metric of overall probabilistic calibration as the mean weighted squared difference of binned predicted probabilities versus the observed frequency of events, $\frac{1}{N}\sum {n}_{k}{\left({\overline{p} }_{k}-{\overline{o} }_{k}\right)}^{2}$, where *N* is the total number of a team’s prediction, *n*_*k*_ is the number of predictions in bin *k* (e.g. between 0.2 and 0.3) with average probability ${\overline{p} }_{k}$, and ${\overline{o} }_{k}$ is the frequency of those predictions being correct. In other words, we assessed if events that were predicted to occur 20–30% actually occurred 20–30% of the time. Our chosen calibration metric corresponds to the reliability term in the Brier score decomposition [35, 36] and has been used to evaluate calibration of another vector-borne disease forecasting challenge [37]. Note that this considers calibration within the single forecast year and provides no information on calibration of models across forecast years.

To assess discriminatory power, we used receiver-operator characteristic (ROC) curve analysis to assess the sensitivity and specificity of the probability of having at least one WNND case in each county. We then calculated the area under the curve (AUC) as the metric for discrimination.

### Regression modeling

We used Bayesian regression modeling to identify high-level modeling approaches and contextual factors of counties associated with variation in skill. To assess the impact of modeling approach, we fitted generalized linear models to all team forecasts and the negative binomial comparison model (April and May versions) using the negative logarithmic score, or surprisal, as the outcome, assuming a Gamma distribution with the inverse link. We used the *stan_glm* function in the rstanarm package (version: 2.21.1, [38]) to fit the models. We assessed associations between surprisal and a suite of model-specific nominal covariates for a team's inclusion of data on climate, human demographics, land use, mosquito distributions/surveillance, and bird/equine infections, and if submissions were updated. To assess county-specific contextual factors, we fitted Bayesian generalized additive models (GAMs) to the ensemble forecasts using the *stan_gamm4* function in the rstanarm package (version: 2.21.1, [38]). We chose the ensemble forecast to capture the overall accuracy of all teams without the variation in performance between teams due to modeling approaches. Contextual factors investigated included environmental factors (e.g. land use, extreme minimum winter temperature, region), history of reported WNND cases (e.g. number of years and pattern of reported cases), and demographics (e.g. population size, population density, population > 65 years old). See Additional file 1: Text S1 for more details on methods, model selection, and a complete list of variables considered.

All analyses were performed with R statistical software (version 4.1.2; [31]).

## Results

Fifteen teams submitted binned probabilistic forecasts for the total number of WNND cases reported in each county using a variety of modeling approaches (see Additional file 1: Text S1 for team information including model details and descriptions and Table S1 for model characteristics). Two teams (13%) included mechanistic model elements while the remainder used completely statistical approaches. Six teams (40%) used Bayesian frameworks for model fitting. We broadly categorized the modeling approaches teams used as machine learning (i.e. random forest, neural network), regression (i.e. maximum likelihood generalized linear models, generalized additive models), hurdle models (i.e. spatio-temporal hurdle models fit using integrated nested Laplace estimation), system of difference equations, or historical case distributions. Across the four submission time points, we received 30 unique forecast submissions (15 initial submissions, 5 teams that updated once, 2 that updated twice, and 2 that updated three times). Some teams used different data sources in different submissions. Across all submissions, 24 submissions (from 11 teams) used climatic data, 22 (from 11 teams) used human demographic data, 9 (from 5 teams) used land-use data, 12 (from 4 teams) used entomological data related to *Culex* mosquito species distributions or WNV infection prevalence in mosquitoes, 2 used data on avian WNV infections (1 team), and 2 used data on equine WNV infections (1 team).

**Fig. 1:**
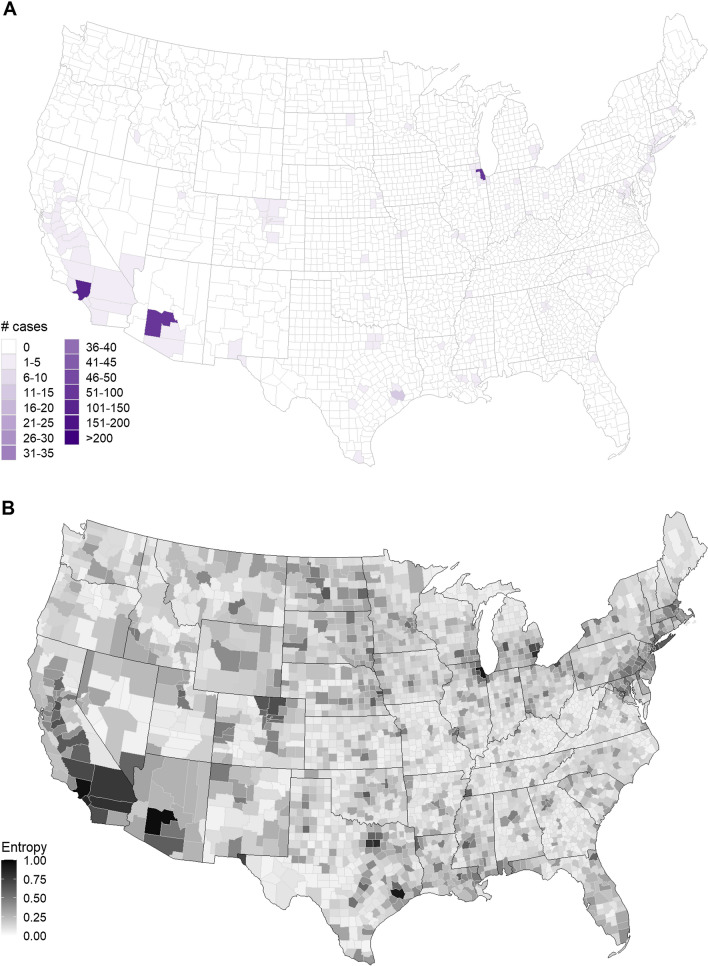
Ensemble forecast with final submissions. **A** Most likely number of WNND cases from and **B** uncertainty (Shannon entropy) of ensemble model forecast. Mean ensemble model built using the last submitted versions of forecasts of all teams and negative binomial model (2000–2019 data). Shannon entropy measures the spread of probability across the binned case counts with a value of zero indicating high certainty in prediction (all probability in a single bin) and a value of one indicating high uncertainty in prediction (probability equally spread across all bins)

The final version of the ensemble model assigned the highest probability to a non-zero bin for 115 counties, with the largest probabilities assigned to high numbers of WNND cases in highly urbanized counties: Los Angeles (CA, bin: 101–150 cases), Maricopa (AZ, bin: 51–100 cases), Cook (IL, bin: 51–100 cases), and Harris (TX, bin: 11–15 cases) (Fig. 1A); the other 111 counties assigned the highest probability to the 1–5 cases bin. The remaining 2993 counties had the highest probability in the ensemble model assigned to the zero-case bin and each team model (final version) assigned the highest probability to the zero-case bin for at least 2222 counties. Uncertainty in ensemble predictions was greatest in more populous counties as well as in the southwest (CA, AZ, NV), in the Great Plains states, along the southern edges of the Great Lakes, and along the northeast coast (Fig. 1B**)**.

Finalized case data for 2020 were released in November 2021 with 559 WNND cases reported in 181 counties. These counts were similar to totals reported annually during 2008–2011 and 2019 (Additional file 1: Table S2). The ratio of reported neuroinvasive to non-neuroinvasive cases was 3.25, the largest reported since 2001 (range for 2002–2019: 0.41–2.43).

**Fig. 2:**
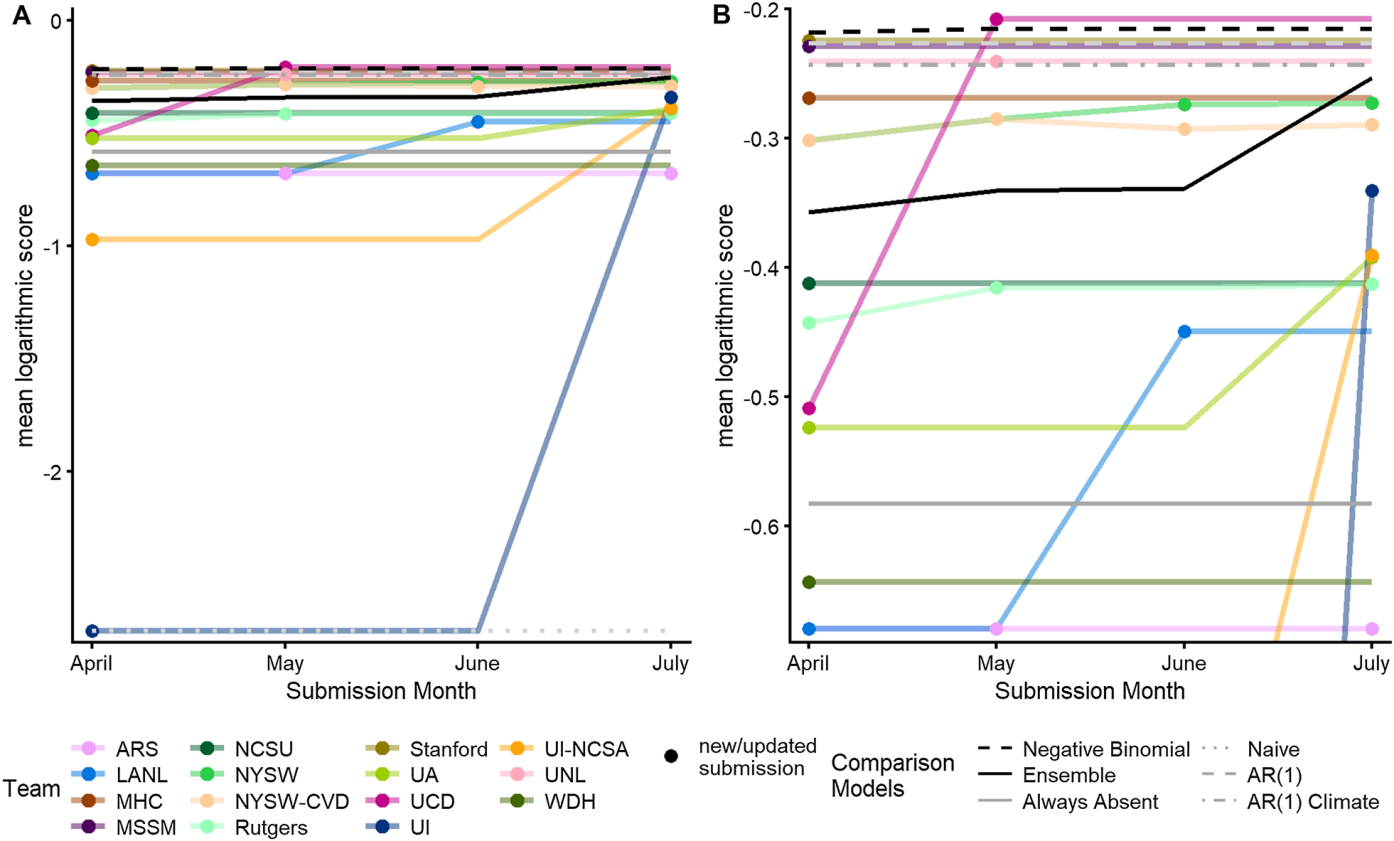
Mean logarithmic score of submissions from teams and comparison models. **A** Full range of mean scores and **B** vertically truncated range to visualize differences in score among top models for each submission time point. If a team did not submit a new forecast at a submission time point, we used the previously submitted forecast to calculate the score (i.e. no variation in score between time points). See Additional file 1: Table S3 for individual forecast mean logarithmic scores

Forecast skill, as measured by logarithmic score, generally increased across the submission time points with updated submissions (Fig. 2, Additional file 1: Table S3). Gains in skill for individual forecasting teams were typically abrupt and occurred at different times, presumably due to acquisition of new contextual data or revisions of modeling approaches. The ensemble forecast, which included all the most recent team forecasts and the negative binomial model at each time point, increased from a mean log score of -0.357 (April) to -0.253 (July), with the largest increase in skill occurring between the June and July submissions likely because of the dramatic improvement in the forecast by *UI*. Three teams (*MSSM, Stanford*, and *UNL*) and the negative binomial forecast consistently outscored the ensemble forecast with four teams *(MHC, NYSW, NYSW-CVD*, and *UCD)* outscoring the ensemble for at least one submission time point. The retrospectively implemented AR(1) and AR(1) Climate models (using mean winter temperature based on historical performance, Additional file 1: Fig S1) also consistently outperformed the ensemble. However, the difference in score between the final forecast for each of those that outscored the ensemble was not statistically significant (*P* > 0.1*,* Additional file 1: Fig S4)*.*

Overall, models based only on historical distributions of cases had relatively high skill. The negative binomial comparison model, AR(1) comparison model, and an empirically weighted distribution (*MSSM*) were in the top five forecasts at each submission time point. Only the final forecast from *UCD* scored higher than the negative binomial model with a difference in mean logarithmic score of 0.007 (*P* = 0.98, Additional file 1: Fig S4).

Comparing high-level modeling approaches and controlling for submission date, we found variation in forecast skill was associated with the inclusion of some types of data (Additional file 1: Table S4). Skill was higher for teams that included climate (0.187, 95% CI 0.174, 0.226) or demographic data (0.335, 95% CI 0.326, 0.361). We found lower skill for forecasts that included land use (− 0.100, 95% CI − 0.124, − 0.031) or *Culex* mosquito geography (estimated ranges or WNV infection prevalence data, − 0.114, 95% CI − 0.142, − 0.048). We did not compare the association of skill with the inclusion of avian or equine WNV disease cases because only one team used each of these data types.

We next analyzed county-specific contextual factors that might be associated with varying forecast skill across modeling approaches by analyzing associations with ensemble forecast skill (Additional file 1: Fig S3). Average skill was highest in counties with mid-sized populations, low historical variation in annual WNND cases (permutation entropy), and relatively moderate winter minimum temperatures (− 10° and 10°F, corresponding to the USDA Plant Hardiness Zones 6a to 7b). For extreme minimum winter temperatures, the ensemble had lower skill at extreme high and low values. For population size, the ensemble had lower skill at large sizes and a nonsignificant relationship at small sizes. Increased variation in interannual historic WNND cases (larger permutation entropy) was associated with decreased forecast skill with a plateau at permutation entropy above approximately 0.7.

**Fig. 3:**
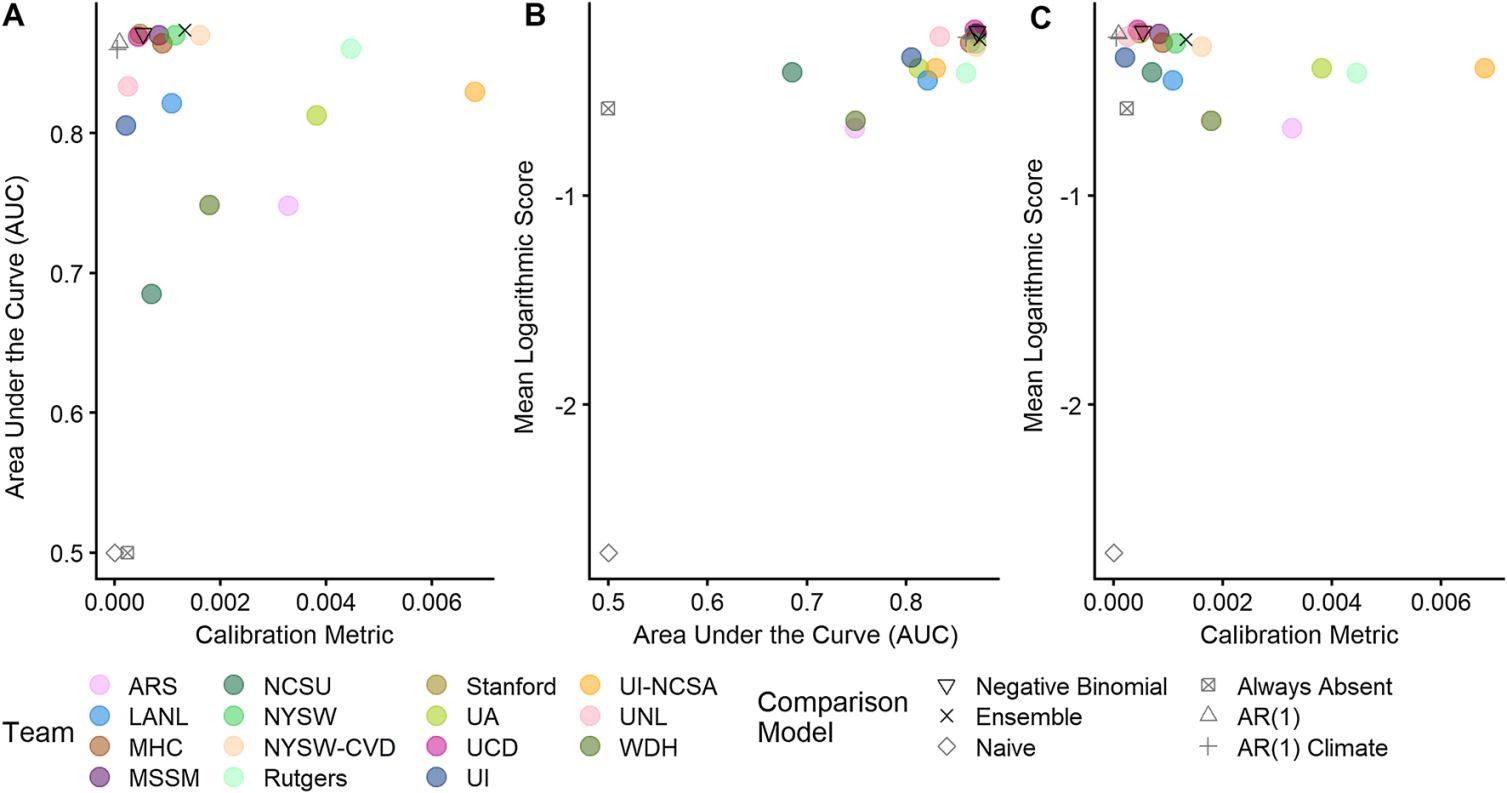
Discrimination, calibration, and mean logarithmic score of final forecasts by teams and comparison models. Area under the curve (AUC) was used to measure a forecast’s ability to discriminate situations with reported WNV cases vs. no cases (AUC of 1.0 would indicate perfect discrimination). Calibration was calculated as the mean weighted squared difference of binned predicted probabilities vs. observed frequency of events (metric of 0 perfectly calibrated). Mean logarithmic score of 0 indicates perfect prediction accuracy. Top-performing models are in the top left (**A**, **C**) or top right (**B**). See Additional file 1: Table S3 and Fig S5-S6 for individual forecast score, calibration, and discrimination

Calibration of forecast uncertainty and the ability to predict whether WNND cases would occur (≥ 1 vs. 0 cases, i.e. discrimination) varied across teams (Fig. 3). Comparing binned forecasted probabilities to observations (Additional File 1: Fig S5), we found that most forecasts were over-confident at lower probabilities and under-confident at higher probabilities. Expectations of the occurrence of cases, especially large numbers of cases, were commonly assigned low probabilities while the expectation of no reported cases was typically highly probable. The forecasts with the best calibration (i.e. reliable specification of probabilities) were those that did not assign any high probabilities (e.g. the naïve forecast), followed by the autoregressive (AR(1) and AR(1) Climate) and negative binomial models. We found that the discriminatory power of forecasts, assessed as the AUC comparing the probability of one or more cases in each county to whether at least one WNND case was reported, also varied widely across teams and comparison models (range of forecast AUC: 0.5–0.875, Additional file 1: Fig S6). The naïve and always-absent comparison models had the worst discriminatory performance, while the ensemble, the negative binomial, the AR(1), the AR(1) Climate forecasts, and several teams (*MHC, MSSM, NYSW, NYSW-CVD, Rutgers, Stanford*, and *UCD*) all had high discriminatory power. The forecasts with the highest overall skill combined good calibration and discrimination.

## Discussion

Reliable early-warning of vector-borne disease outbreaks could offer new opportunities for effective prevention and control through targeting control to high-risk areas. For WNV, such an early-warning system would identify spatial and temporal periods of high-risk weeks to months prior to the onset of risk, enabling effective proactive response. We performed a multi-model evaluation of probabilistic forecasts for the total WNND cases reported by county in the contiguous US and Washington DC in 2020. The comparison of forecast performance elucidated the current predictive capacity of WNND on this spatial and temporal scale and avenues for improvement.

Although the COVID-19 pandemic caused dramatic changes in human behavior and challenges for health systems in 2020, it is not clear that the occurrence and reporting of WNND cases changed dramatically. The reported total number of WNND cases was similar to prior years with relatively low case numbers. The ratio of reported WNND to non-neuroinvasive cases for 2020 increased substantially, to the highest level since 2001, indicating likely under-detection and -reporting of non-neuroinvasive cases. However, it remains unclear what impact COVID-19 may have had on human behavior and resulting exposure to WNV, treatment-seeking by infected individuals, or physicians’ diagnosis and reporting of WNV disease.

Overall, simple models based on historical WNND cases (i.e. the negative binomial model) generally scored better than more complex models, combining discriminatory power and calibration of uncertainty. Only one team (*UCD*) had higher forecast skill than the negative binomial forecast model, and only by a small, nonsignificant margin. One explanation for the relatively strong performance of the negative binomial model is that the historical case distributions reflect the ecological differences across counties and therefore capture most of the inherent spatial variability in WNV transmission. Incorporating additional contextual factors explicitly might not necessarily improve prediction accuracy despite their importance. Also, matching case locations in space and time with available environmental data can introduce uncertainty in model predictions that consider environmental data on top of historical WNV data. For example, WNND data were available on the county-annual scale while environmental data were available at much finer spatial and temporal resolutions. Thus, decisions on aggregations or summaries of environmental data cannot fully capture the particular sequence of conditions precipitating zoonotic transmission.

Regression to identify modeling approaches associated with variation in forecast skill confirmed an increase in score for later submissions after accounting for other differences. Changes in later forecast submissions were attributed largely to integration of updated data rather than changes in forecasting methods, so this score improvement highlights the value of including updated covariate data (e.g. reported updates included using recent weather data, newly released 2019 WNV data, and additional demographic data). Although we could not discern the relative contribution of each update on the change in score due to heterogeneity in the type of changes and number of submissions across teams, recent weather data appeared to have played some role in improving the predictive accuracy of forecasts. Improving access to real-time data streams could therefore improve predictive accuracy [27, 39]. Moreover, these updates occurred before the majority of WNND cases were reported, indicating that although forecasts that provide early warning during the spring can allow for greater lead times for preventative actions, later updates that provide early detection of risk—even after some cases have begun to occur—could provide additional value [27]. From a practical standpoint, shifting forecast submission deadlines by several days later could facilitate incorporating monthly aggregated data from the prior month when available.

The limited number of submissions prevented us from fully assessing the relative performance of different modeling approaches as models used different data inputs in addition to different methods. While the broad classifications we used provide some insight on general forecast skill, we could not assess the performance of specific model constructions because they varied in both methods and covariates included. It could be of interest to identify variation in predictive performance because of specific model constructions to guide the development and refinement of WNV prediction.

We found the inclusion of estimated mosquito distributions or mosquito surveillance data reduced forecast skill on average. This result seems counterintuitive because the importance of key mosquito vectors and the relationship between entomological indicators of risk and WNV activity is clear [9, 10, 40–43]. One explanation is that mosquitoes are much more widespread than WNND cases, so it is difficult to discriminate counties with intense enzootic transmission without human involvement. An alternative explanation is that this finding might reflect model-specific limitations in how the data were incorporated or limited quality or availability of national datasets on mosquito distributions or entomological surveillance. Current distribution maps date back to the 1980s [44, 45] with an update in 2021 using habitat suitability modeling [46]. Although the updated maps have increased spatial definition compared to earlier estimates, these distributions indicate relative habitat suitability rather than presence or absence. One publicly available surveillance database, ArboNET, maintains data on human disease and infections among presumptive viremic blood donors, veterinary disease cases, mosquitoes, dead birds, and sentinel animals for a variety of arboviruses. However, nonhuman arboviral surveillance is voluntary with large variation in spatial and temporal coverage between jurisdictions, and reported data are often incomplete [47] reducing the predictive utility of the database.

The ensemble forecast had a higher forecasting skill (average logarithmic score) than most team forecasts, with better discriminatory power (ability to differentiate having at least one case) than any team forecast and better calibration (reliable uncertainty specification) than most. Previous forecasting efforts for influenza, dengue, and COVID-19 [37, 48–50] demonstrated that ensemble approaches capitalize on the strengths of diverse models and balance uncertainty across modeling approaches to produce robust predictions. This general finding was replicated here with the ensemble performing in the top third of forecasts. However, we also found a simple model based on historical data alone substantially outperformed both the ensemble and majority of team forecasts at every submission date for the 2020 Challenge. This indicates that even the strengths of a multi-modeling approach were not sufficient to improve prediction beyond historical trends for this year. There are several potential ways to improve the ensemble in the future. With predictions for previous years it would be possible to generate weighted ensembles that could improve performance. Weighted ensembles based on regional performance could also potentially leverage differences in forecast skill for different ecological zones. Alternative approaches to generating ensembles from component models such as linear pools from cumulative distribution functions which could be approximated from binned forecast probabilities could also be fruitful [51, 52].

We found that heterogeneity in historic WNV cases had a significant impact on variation in forecast skill, and unsurprisingly, forecasts scored worse in locations of high historic heterogeneity. Improvement in forecast skill for these locations would likely be the most useful for vector control and public health officials, but the high variability also represents a significant challenge to forecasters.

Other intrinsic differences between counties associated with lower forecast skill could highlight areas that need improvement. By identifying local drivers in counties with relatively large populations and hotter or colder winters, forecast skill could be improved in these circumstances. For example, the ecological setting (i.e. *Culex* species present, composition of avian community, and climate) would vary substantially between counties with “hot” or “cold” winter extremes and different drivers may need to be considered in each. Also, factors might interact together to impact zoonotic transmission, but due to the limited data and limited number of forecasts available for analysis, we were unable to investigate these.

Calibration across teams indicated other avenues for improving prediction. Overall, teams over-predicted the probability that cases would occur while correspondingly underestimating the probability that cases would not occur. Overestimating the probability of disease cases could lead to better preparedness but could also result in allocation of resources that are not ultimately needed. Moreover, repeated instances of non-events could lead public health officials or the public to doubt the accuracy of such forecasts. A forecast with demonstrated calibration is not immune to this type of perception but would be able to demonstrate over time or across locations that an 80% chance of an outbreak still results in no outbreak 20% of the time. Further work on refining calibration and identifying any relationship of modeling approach and calibration could improve the reliability and usability of forecasts.

The identification of climate factors predictive for WNV activity needs further refinement. Our analysis of modeling approaches indicated that teams that included climate data scored better than those that did not. However, the data source, climatic variables (e.g. minimum temperature, maximum temperature, total precipitation, variance in precipitation, Palmer Drought Severity score, dewpoint, soil moisture, anomalies in temperature or precipitation), and aggregation of the climate variable (e.g. number of days above or below a threshold; weekly average; average of 1–12 months; lagged values up to 3 years) varied widely among teams (Additional file 1: Text S1). It should be noted that all climate data included in models was lagged to some extent in relation to the predicted annual totals. Due to heterogeneity among teams and the limited number of total forecasts, we could not identify the most predictive subset of climatic factors and appropriate spatial and temporal aggregations or lags nor the potential importance of variation in data quality among data sources. Similarly, the addition of any seasonal climatic variable in the autoregressive modeling framework when selecting the baseline climate model reduced the forecast skill relative to the AR(1) model (Additional file 1: Fig S1). However, this model, which used a single climate variable nationally on a subjectively prescribed 3-month season, could not capture spatial variation in climatic zones. Previous studies have also demonstrated challenges in identifying a single environmental driver for predicting WNV activity [53–57]. The essential role of climate in WNV transmission likely varies substantially across different ecological areas, with geographic heterogeneity in which combination of environmental factors, avian populations (composition and seropositivity), and mosquito species drive local transmission.

The forecasts generated here provide some important insight on the challenges with current capabilities and opportunities for improvement, but also on potential uses. As in other forecasting efforts, an ensemble was more accurate than many of the individual component forecasts. However, in this case, a model based on historical data had more forecast skill and could be considered as a benchmark for a national-scale early warning system even though the current best indicator of high risk is a past history of larger outbreaks. The use of heuristic principles, like historic outbreaks, can be useful, but sometimes leads to severe and systematic errors [58]. Early indications of high risk can support preparedness across scales, such as resource planning and allocation at the state or local scale. Forecasts at finer spatio-temporal resolution (e.g. 2-week forecast on the neighborhood scale) could be even more useful to directly guide effective vector control within counties within seasons [27]. Additional targets like onset or peak week of transmission could also guide vector control activities. There might also be opportunities to frame and communicate forecasts more effectively. Here, we have focused on binned probabilities of different case numbers. However, forecasts could also be framed as the probability of above average incidence or predicted range of case numbers (e.g. a 90% prediction interval) that might be actionable in different ways.

## Conclusions

The 2020 WNV Forecasting Challenge highlighted the current state of large-scale, early-warning prediction capacity for WNND cases in the US. Simple models based on previous WNND cases generally performed better than more complex forecasts. The forecasts evaluated therefore indicate that historical incidence provides a relatively reliable indicator of future risk, but substantial uncertainty remains, and future models can build upon findings here to improve forecasting as well as providing insight on the probability that the next season will be different from previous seasons. Among models using additional data, inclusion of climate or human demographic data was associated with higher skill, while inclusion of mosquito or land use data was associated with lower skill. These differences indicate that WNV forecasts can benefit by considering location-specific historical data and incorporating additional covariates with caution. Forecast skill was also associated with intrinsic differences among counties, with lower skill in counties with relatively large populations, “cold” or “hot” winters, and high variability in yearly case counts. High case count variability likely indicates counties that are intrinsically more difficult to predict, but there may be opportunities to specifically improve predictions for areas with large populations and low or high winter temperatures. Most forecasts, including the highest skill forecasts, also showed patterns of calibration that could potentially be improved. In addition to improved forecast models, increased data collection, data sharing, and real-time data access [e.g. meteorological observations, avian immunity to WNV, mosquito surveillance (abundance and infection rates), mosquito control activities] may support improved predictions. These findings lay the foundation for improving future WNV forecasts.

## Supplementary Information

**Additional file 1: Text S1.** Appendix. **Fig. S1.** Mean logarithmic score of AR(1) models. **Fig. S2.** Coefficients in AR(1) Climate model. **Fig. S3.** Smooth functions of contextual factors associated with variation in forecast skill. **Fig. S4.** Significance of difference in mean logarithmic score between forecasts. **Fig. S5.** Calibration of forecasts by teams and comparison models. **Fig. S6.** Receiver-operator characteristic (ROC) curves for forecasts by (A) teams and (B) comparison models. **Table S1.** Model characteristics and classes of covariates included in each team’s model. **Table S2.** Reported West Nile virus neuroinvasive and non-neuroinvasive disease cases (2000–2020). **Table S3.** Mean logarithmic score for each team’s submitted forecast and six comparison models. **Table S4.** Regression coefficients from Bayesian generalized linear model for modeling approaches associated with variation in skill.

## Data Availability

The datasets used and/or analyzed during the current study are available are available in the WNV-forecast-project-2020 repository, https://github.com/cdcepi/WNV-forecast-project-2020.

## Abbreviations

CDC: Centers for Disease Control and Prevention
WNND: West Nile virus neuroinvasive disease
WNV: West Nile virus
