# Supplementary material for: Evaluation of an open forecasting challenge to assess skill of West Nile virus neuroinvasive disease prediction
Source: Parasit Vectors. 2023 Jan 12;16:11. doi: 10.1186/s13071-022-05630-y (PMC9834680; doi:10.1186/s13071-022-05630-y)
Supplement: Supplementary file 1 — Additional file 1: Text S1. Appendix. Fig. S1. Mean logarithmic score of AR(1) models. Fig. S2. Coefficients in AR(1) Climate model. Fig. S3. Smooth functions of contextual factors associated with variation in forecast skill. Fig. S4. Significance of difference in mean logarithmic score between forecasts. Fig. S5. Calibration of forecasts by teams and comparison models. Fig. S6. Receiver-operator characteristic (ROC) curves for forecasts by (A) teams and (B) comparison models. Table S1. Model characteristics and classes of covariates included in each team’s model. Table S2. Reported West Nile virus neuroinvasive and non-neuroinvasive disease cases (2000–2020). Table S3. Mean logarithmic score for each team’s submitted forecast and six comparison models. Table S4. Regression coefficients from Bayesian generalized linear model for modeling approaches associated with variation in skill. [file 13071_2022_5630_MOESM1_ESM.docx]

**Additional File 1 contains:**

**Text S1**

**Fig S1-S6**

**Table S1-S4**

**Text S1. Appendix**

**Forecast methods**

The following are summarized model descriptions for each team’s approach to predicting binned probabilities of case numbers. Descriptions are organized alphabetically by team name which reflects the institutional affiliation of the team lead (indicated in square brackets for the first entry of each team’s description). Each team submitted a brief description of their approach (e.g., modeling framework, datasets used, covariate development) and any updates made to model methodology between submissions (see the project’s GitHub repository for original model descriptions; https://github.com/cdcepi/WNV-forecast-project-2020). Teams reserved the right to publish their own research and we included citations for those who have published their approach. High-level model characteristics are summarized in Table S1 and mean logarithmic scores in Table S2.

*ARS [Agricultural Research Service, USDA]:* A Bayesian hierarchical model was used to develop a two-part, spatiotemporal model (conditional autoregressive model). The first level predicted non-neuroinvasive counts and the second level, using these counts, predicted neuroinvasive counts. Other input variables were median household income, force of infection, proportion of population over 54 years of age, geographic county area, competent avian host richness, maximum temperature, total precipitation, and mosquito and avian detections reported to ArboNET. Starting in May, Native American population variables (population >18 years of age and area of tribal lands) were included. The model included two, separate spatial effects to control for spatial bias/error within each model level, as well as a third “shared” spatial effect to account for the spatial relationship between neuroinvasive and non-neuroinvasive counts. The reported case year was used to model a non-linear time trend for the period of record and a space-time interaction term (Year*County) was added to quantify the relationship between the spatial and temporal trends. See [1] for further details.

*LANL [Los Alamos National Laboratory]:* Forecasts were made using the mean number of cases from 2005-2018, rounded to the nearest whole number. The bin including the mean value was assigned a probability of 0.797 and all other bins were assigned a probability of 0.0145.

*LANL (updated):* Forecasts were made considering the mean and variance of historical case counts (2005-2019). If the mean of historic counts was greater than or equal to the variance, probabilities were generated from random sampling of a Poisson distribution with mean of the historical mean case count. If the mean of historic counts was less than the variance, bin probabilities were generated from random sampling of a negative binomial distribution fitted to the bins containing historical case counts. If no cases were reported from 2005-2019, a probability of 0.85 was assigned to the first bin [0,1); 0.1 to the second bin [1,6); 0.05 to the third bin [6-11); and 0.00 to the remaining bins.

*MHC [Mount Holyoke College]:* Forecasts were made using a zero-inflated Poisson regression model containing tensor product B-splines for location. The probability of the Poisson component was obtained as inverse-logit{population * spline(latitude, longitude)} and the mean of the Poisson distribution was population * exp{spline(latitude, longitude)}. The model was fitted using Markov Chain Monte Carlo sampling.

*MSSM [Icahn School of Medicine at Mount Sinai]:* Forecasts were generated from an optimally weighted historical empirical distributions of cases from 2003-2018. Empirical distributions were generated at the county, state, and national scale. Empirical distribution weights were determined by identifying the optimal forecast skill using a leave-one-out validation from 2003 to 2018. The optimal weighting for the county, state, and national distributions were 75%, 15%, and 10%, respectively.

*NCSU [North Carolina State University]:* Forecasts were made using a system of differential equations (weekly counts of susceptible and infected individuals per county from 2000-2018). The rate of transition from susceptible to infected classes was determined for each county. Using the covariates of interest (social vulnerability index and smoothed daily minimum temperature), a log-transformed linear regression for the transition rate parameter was fitted using a Delayed Rejection Adaptive Metropolis (DRAM) algorithm. Forecasts were made using the posterior distributions from the regression analysis and covariates for each county for 2020.

*NYSW [New York State Department of Public Health, Wadsworth Center]:* Similar to [2], predictions were made using a quantile random forest model with mosquito ranges, air conditioning use, population size, weather data (temperature, precipitation, relative humidity, and vapor pressure deficit), soil moisture, and historical case counts as input predictor variables. Weather and soil moisture data were aggregated for Jan-Mar for the April submission and then added as monthly variables for each additional submission, based on data availability. A mean importance score from the model was calculated and all variables below the mean importance were dropped and the model was refit.

*NYSW-CVD [New York State Department of Public Health, Wadsworth Center]:* Predictions were made using the same quantile random forest model as in *NYSW*, but with a scaling factor applied to increase or decrease the forecasted risks based on observed changes in human behavior related to COVID-19 and resulting in changes to human-mosquito interactions. Risk adjustment was based on changes in human outdoor activity from the TickApp [3] for 2020 relative to a 2018 and 2019 baseline. Risk adjustments were applied for May, June, and July submission (identical submission for *NYSW* and *NYSW-CVD* for April).

*Rutgers [Center for Vector Biology, Rutgers University]:* A Bayesian model was fitted to weighted binned case counts using climatic (climatic NOAA region, previous year precipitation, minimum temperature in January), land use (% suburban and agricultural areas each county), lagged WNV case counts (2-year lag), average number of WNV cases in the neighboring counties, and population density as predictors. Highly correlated predictors were removed prior to analysis.

*Stanford [Stanford University]:* Forecasts were generated using a boosted classification tree (Hessian-based boosting) fit to the categorical bin outcomes. Input parameters included modeled mosquito distribution estimates, temperature-dependent R0 estimate, climate data (temperature, precipitation, Palmer Drought Index Severity score), WNV variables (historic county case average and totals, historic state total cases, and adjacent county average and total cases), equine cases, demographics (total population size and proportion in rural, micropolitan, and metropolitan areas), and land cover. Training data to select final model structure (i.e., boosted regression or classification tree fit to either counts or categorical bins) used 2000-2016 data to predict 2017-2018 counts. The evaluation metric for count outcomes was Poisson negative log-likelihood and multiclass log loss for categorical outcomes.

*UA [University of Arizona]*: The model used a neural network architecture with three dense layers, a batch normalization layer and an output layer. Input variables fed into the neural network were the following climatic variables: mean precipitation, mean maximum temperature, mean minimum temperature, standard deviation of precipitation, standard deviation of maximum temperature, and standard deviation of minimum temperature.

*UA (updated)*: The models used a system of two 1D convolutional neural networks to generate binned predictions. The first model was a binary classifier trained to predict whether a county will have WNV neuroinvasive cases or not. Samples with a probability of zero cases > 0.75 were fed into a second model, a categorical classifier trained to predict the probability of each bin used in the Challenge. Variables in both models included USDA Plant Hardiness Zones, population size, and the climatic variables listed above as well as number of days with minimum or maximum temperatures at specific thresholds (i.e., <13°, 13°-21°, 21°-38°, and >38°C).

*UCD [University of California, Davis]:* Forecasts were modeled through a spatio-temporal Bayesian-inference hurdle model with two likelihoods, a binomial likelihood for the occurrence of WNV neuroinvasive cases, and a truncated negative binomial for the number of cases. Spatially (Besag-York-Molliѐ 2 model) and temporally (random walk of second order) structured effects were shared between the likelihoods. Model selection of included variables (i.e., temporally-matched climate, land use, major bird host relative abundance, one-year lagged climate, and human demographics) was based on the logarithmic score of the Conditional Prediction Ordinate (CPO).

*UI [University of Illinois]:* The forecast consisted of equal probability assigned to each bins, akin to the naive model in our comparison models.

*UI (updated):* Forecasts were made using a zero-inflated Poisson regression model. The model first used logistic regression to account for counties with no historic neuroinvasive cases and then ran a Poisson model to predict case counts for counties reporting at least one previous neuroinvasive case. Input variables included land cover, climatic variables (precipitation, temperature, and dewpoint), and socio-demographic factors (average age per census block, race and ethnicity, and average household income). Covariate inclusion criteria was conducted via forward selection and the final models were selected by Akaike Information Criteria (AIC).

*UI-NCSA [National Center for Supercomputing Applications, University of Illinois]:* Prediction was accomplished by developing a Long Short Term Memory (LSTM) recurrent neural network. To include the temporal effect, the input parameter involved the past 5 years data to predict the current year. For each prior year used, the features included were weather (temperature, precipitation, humidity, and Gini index of precipitation), WNV variables (total WNV cases, neighboring county average WNV cases, and an indicator of outbreak years), and county type (rural or urban).

*UI-NCSA (updated):* Forecasts used a naive random forest where probabilities per bin were calculated on the voting results of the trees in the random forest. Input parameters included WNV data (lagged case counts, neighboring county average counts, and average state-level counts), weather (temperature, precipitation, humidity, and Gini index of precipitation), and county type.

*UNL [University of Nebraska-Lincoln]:* As previously published [4], forecasts used a generalized additive model with thin-plate splines for non-parametric modeling of distributed lags of drought and temperature data, using restricted maximum likelihood estimation with a log link and negative binomial distribution. Population (natural log) was included as an offset. Sum-to-zero contrasts were used as random effects to capture intra-class correlation of years. Lagged temperature and precipitation variables, lagged up to 24 months, were included.

*WDH [Wyoming Department of Health]:* Forecasts were made using a Bayesian, hierarchical generalized additive model that used spatio-temporal smooths and county-level varying effects to extrapolate potential WNV cases in 2020 based on previous trends. County populations were used as an offset. The model assumed a negative binomial likelihood, with the shape parameter having county-level varying effects.

**Regression models**

We compared logarithmic scores using Bayesian regression models to estimate the conditional distribution of transformed scores given specific sets of variables potentially related to forecast skill (contextual factors or modeling approach). For the analyses, we converted the logarithmic scores to surprisal values (-log(*p_i_*)) by changing the sign, thus transforming the outcome variable to a continuous positive value that can be approximated with a Gamma distribution and is on the same scale as the original scores. The truncation of log scores we used to prevent scores of negative infinity (i.e., truncated to a maximum of -10) introduced an artificial density of scores at the corresponding surprisal value. However, the Bayesian paradigm views observations as uncertain and the Markov Chain Monte Carlo sampling distributed these observations across a wide range of scores.

We used Bayesian regression models to assess the effects of multiple variables on the Gamma-distributed surprisal scores (inverse link). For the investigation of modeling approaches, we used Bayesian generalized linear models (GLMs) fitted with Stan (http://mc-stan.org/), using the *stan_glm* function in the rstanarm package (version 2.21.1; [5]). For the investigation of county-level contextual factors, we used Bayesian generalized additive models (GAMs) to capture the nonlinear associations with the variables and outcome. We also fitted these GAMs with Stan using the *stan_gamm4* function in the rstanarm package with default priors. For each regression, we ran four chains with a burn-in of 500 samples, and then collected an additional 500, resulting in 2,000 samples across the four chains. All models were checked for convergence. To compare regression models in each investigation, we used leave-one-out cross validation with Pareto-smoothed importance sampling to estimate the expected log pointwise predictive density (ELPD) [6] as well as root mean squared error (RMSE) and mean absolute error (MAE).

To assess modeling approach associated with variation in skill, we included 0/1 coded variables indicating the inclusion of covariates related to climate, human demographics, land use, mosquito distributions/surveillance, and bird/equine infections. We determined covariate inclusion from model descriptions submitted by each team and fitted the regression models using all team’s forecasts. We could not investigate the impact of including avian or equine cases as only one team each included variables for avian or equine cases. Also, due to the heterogeneity in modeling approaches and relatively small number of submissions, we did not investigate variation in score related to modeling approach used (e.g., Bayesian, maximum likelihood generalized linear or additive regression, machine learning, historical incidence). Regression coefficients for the final model are presented in Table S3.

To assess county-level contextual factors associated with variation in skill, we included variables related to a county’s environment, history of neuroinvasive West Nile virus (WNV) disease cases, and human demographics. For environment variables, we included USDA Plant Hardiness Zones [7], average extreme minimum winter temperature, proportion of the county’s area with specific land use types (i.e., crops, urbanization, and wetlands), and region and division (retrieved from the ‘state’ dataset in R, [8]. We calculated land use variables using the 2011 National Land Cover Database [9], grouping all “Developed” classes as “urban”, the “Cultivated Crops” class as “crops”, and the “Emergent Herbaceous Wetlands” class as “wetlands” for analysis. For WNV history variables, we included average and median annual incidence, average and median annual cases, total number of cases, number of years with ≥1 case, first year cases were reported, and heterogeneity of cases across years. To quantify interannual heterogeneity, we calculated permutation entropy, a measure of complexity of a dynamic system [10] where normalized values close to 0 indicate a regular, deterministic timeseries while values closer to 1 indicate a noisy, random timeseries. When calculating permutation entropy, we used an embedding dimension of 3 based on the length of the historical WNV case data. For demographic variables, we included total population size, population >65 years, proportion of the population >65 years, and population density (people/km^2^), using data from the United States Census Bureau [11].

To investigate contextual factors, we fitted regression models to the ensemble forecast with the final submitted versions of forecasts. We began by fitting univariate models and selected the models with the highest ELPD from each category (i.e., environment, WNV history, and demographics) to begin multivariate model selection. Using this base model, we used forward selection to reach the final model with the largest ELPD. Smooth functions for the variables are presented in Fig S3.

**Development of autoregressive modeling framework**

We used an autoregressive model of order 1 (i.e., an AR(1) model) as the framework to build a baseline climate model. To capture variation in years, we iteratively fit a series of AR(1) models to logged case counts (i.e., ln(cases+1)) and predicted the probability distribution of reported cases for the following year, binned in the same manner as the WNV Challenge forecast submissions. We first fit models to data from 2005-2010 and predicted 2011 cases. Then, we used data from 2005-2011 to predict 2012 cases and so forth until we used 2005-2019 data to predict 2020 cases (10 timeframes). We used data from 2005 onward to limit the influence of invasion dynamics on fit relationships. If no cases were reported in the timeframe used to train the model, we assigned all the probability to the zero-case bin.

For each county and prediction year (2011-2019), we fitted an AR(1) model plus 18 AR(1) Climate models which each included a single seasonal climatic variable. We considered seasonal precipitation accumulation and mean or minimum temperature (three climate variables) due to the known association of these factors with mosquito population and WNV transmission dynamics. We defined seasons as three-month periods for winter (Dec-Feb), spring (Mar-May), summer (Jun-Aug), and fall (Sep-Nov), using climatic data from the previous winter to current spring (six seasons) to predict case distributions (e.g., winter, spring, summer, and fall 2017 and winter and spring 2018 for predicting total 2018 cases). We fitted all models using the *arima* function in the stats package [8].

We used logarithmic scoring to rank the performance of the autoregressive models (­*n* = 19, Fig S1) and selected the model with the seasonal climate variable which had the smallest mean score across all counties and prediction years as the AR(1) Climate model (i.e., AR(1) with mean winter temperature). We updated this model with corresponding mean winter temperature (Dec 2019-Feb 2020) and predicted 2020 case counts. The fitted coefficients for this final AR1(1) Climate model are presented in Fig S2. We also used the AR(1) model without any climate variables as a comparison model in evaluating the submitted WNV Challenge forecasts.

**References**

1. Humphreys JM, Young KI, Cohnstaedt LW, Hanley KA, Peters DPC. Vector surveillance, host species richness, and demographic factors as West Nile disease risk indicators. Viruses. 2021 May 18;13(5):934.

2. Keyel AC, Elison Timm O, Backenson PB, Prussing C, Quinones S, McDonough KA, et al. Seasonal temperatures and hydrological conditions improve the prediction of West Nile virus infection rates in *Culex* mosquitoes and human case counts in New York and Connecticut. PLOS ONE. 2019 Jun 3;14(6):e0217854.

3. Fernandez MP, Bron GM, Kache PA, Larson SR, Maus A, Gustafson Jr D, et al. Usability and feasibility of a smartphone app to assess human behavioral factors associated with tick exposure (the Tick App): Quantitative and qualitative study. JMIR Mhealth Uhealth. 2019 Oct 24;7(10):e14769.

4. Smith KH, Tyre AJ, Hamik J, Hayes MJ, Zhou Y, Dai L. Using climate to explain and predict West Nile virus risk in Nebraska. Geohealth. 2020;4(9).

5. Goodrich B, Gabry J, Ali I, Brilleman S. rstanarm: Bayesian applied regression modeling via Stan. 2020. R package version 2.21.1. Available from: https://mc-stan.org/rstanarm

6. Vehtari A, Gelman A, Gabry J. Practical Bayesian model evaluation using leave-one-out cross-validation and WAIC. Statistics and Computing. 2017;27(5):1413–32.

7. Agricultural Research Service U.S. Department of Agriculture, Oregon State University. USDA Plant Hardiness Zone GIS datasets. 2012. Available from: https://prism.oregonstate.edu/projects/plant_hardiness_zones.php

8. R Core Team. R: A language and environment for statistical computing. Vienna, Austria: R Foundation for Statistical Computing; 2021. version 4.1.2. Available from: https://www.r-project.org/

9. Multi-resolution characteristics consortium. NLCD 2011 land cover (CONUS). 2011. Available from: https://www.mrlc.gov/data/nlcd-2011-land-cover-conus

10. Henry M, Judge G. Permutation entropy and information recovery in nonlinear dynamic economic time series. Econometrics. 2019 Mar 1;7(1).

11. United States Census Bureau. Annual county and resident population estimates by selected age groups and sex: April 1, 2010 to July 1, 2019. County population characteristics: 2010-2019. 2021. Available from: https://www.census.gov/data/tables/time-series/demo/popest/2010s-counties-detail.html

12. Centers of Disease Control and Prevention. West Nile virus disease cases and deaths reported to CDC by year and clinical presentation, 1999-2020. Final cumulative maps & data for 1999-2020. 2021. Available from: https://www.cdc.gov/westnile/statsmaps/cumMapsData.html#three


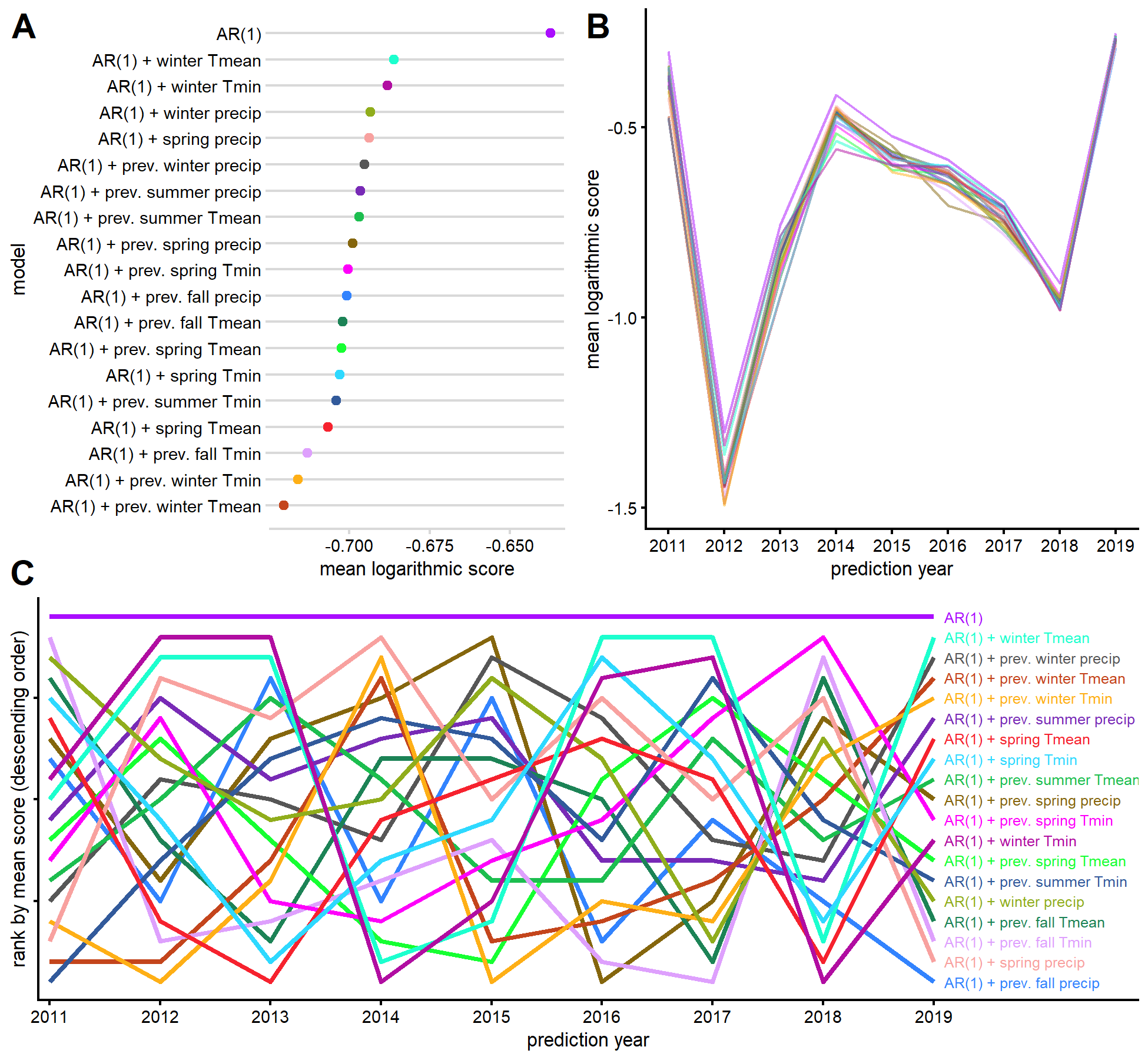
**Fig S1**. **Mean logarithmic score of AR(1) models.** A) Mean score calculated for all prediction years (2011-2019) and counties (*n* = 3,108). B) Mean score for each model by prediction year. C) Ranking of models based on mean logarithmic score by prediction year with the best scoring model on top. Smaller scores indicate better predictions in A and B. Tmean = mean temperature; Tmin = minimum temperature; precip = total precipitation.

**
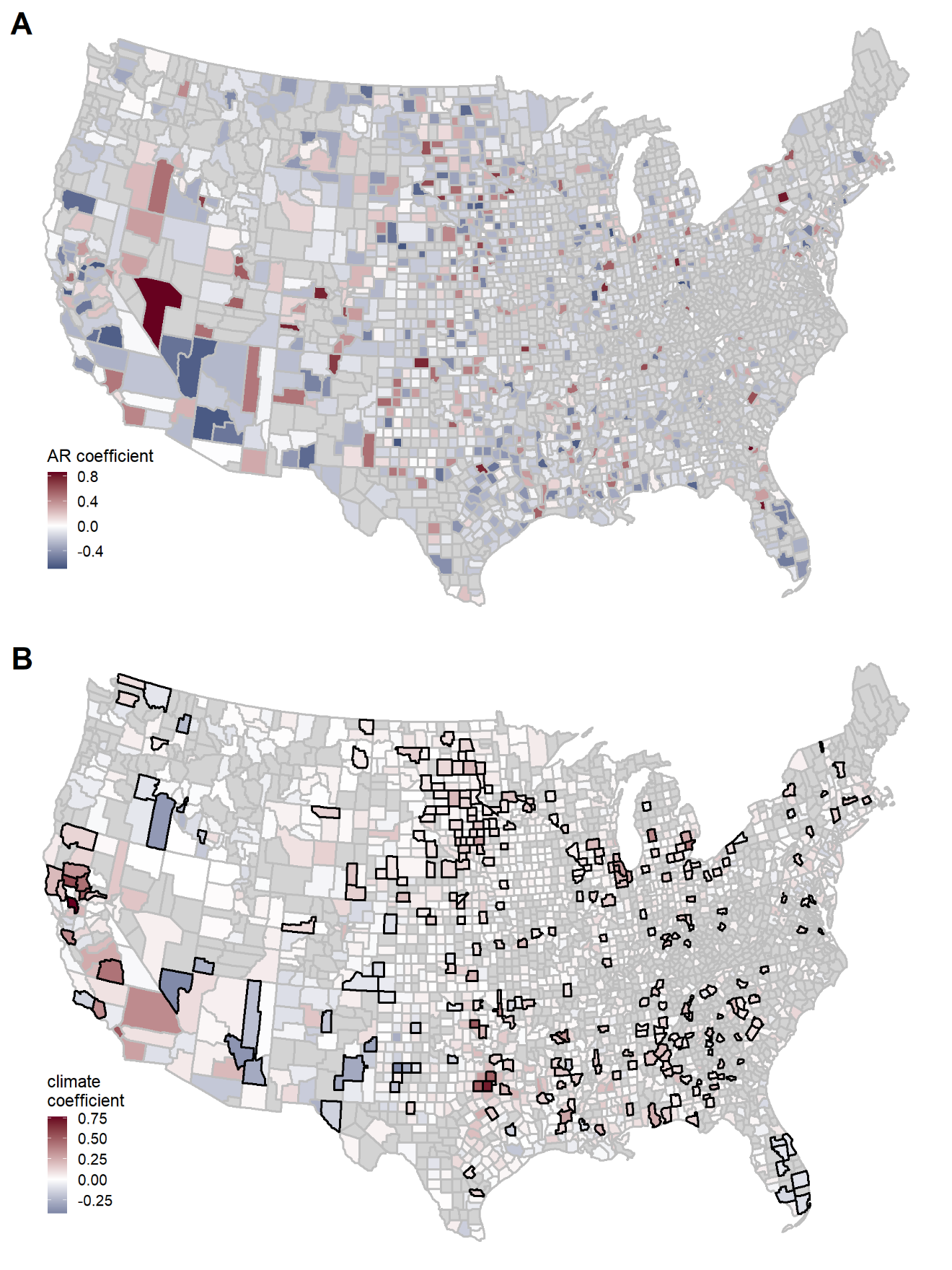
**

**Fig S2. Coefficients in AR(1) Climate model.** A) Autoregressive (AR) and B) climate variable (mean Dec 2019-Feb 2020 (winter) temperature) from the first-order autoregressive model fitted to each county. Counties which never reported neuroinvasive cases (2005-2019) colored grey. Significant coefficients for the climate variable (Wald test, *P* < 0.05) outlined in black in panel B.


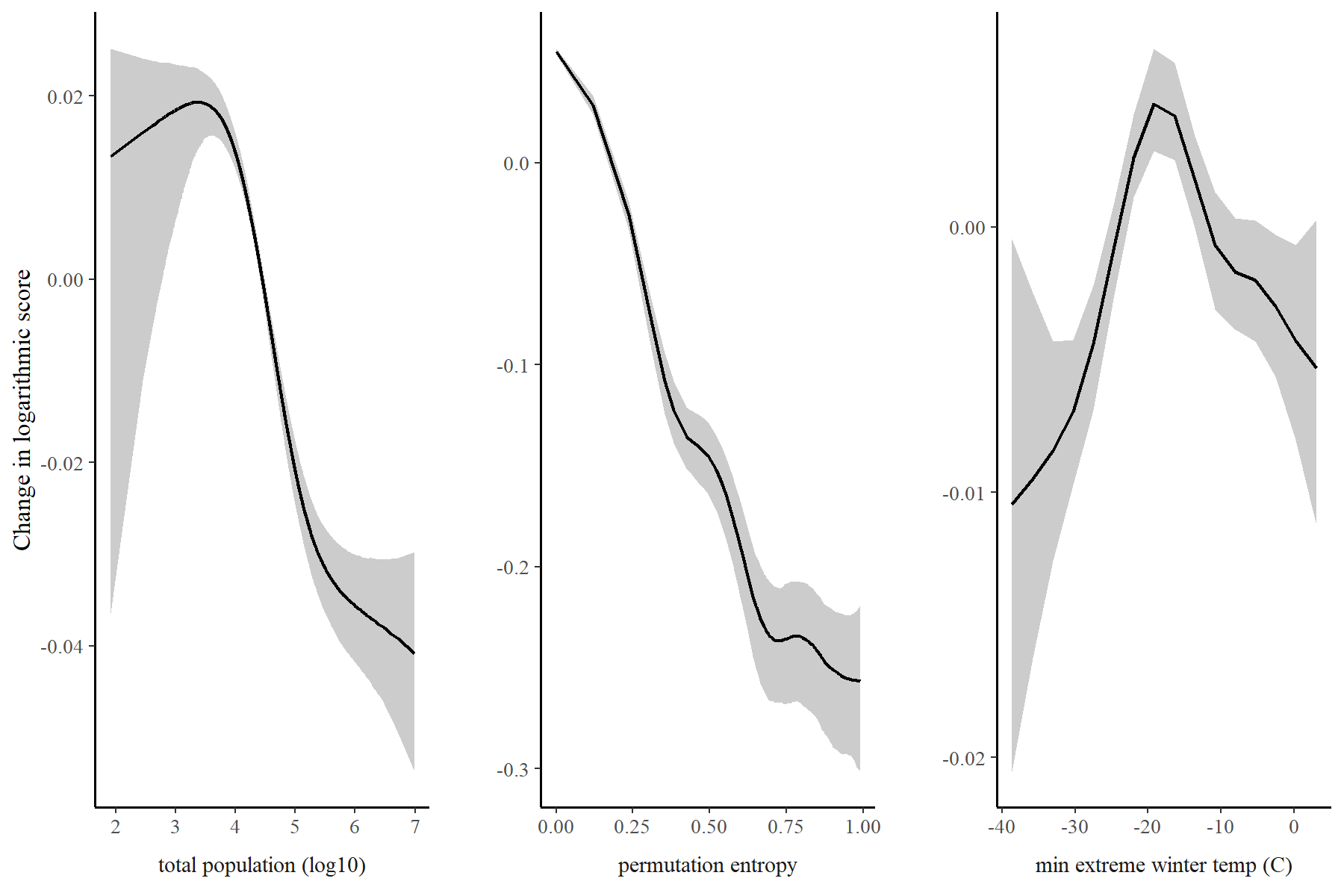


**Fig S3. Smooth functions of contextual factors associated with variation in forecast skill.** Smooth functions from Bayesian generalized additive model of surprisal with ensemble forecasts. Median (solid line) and 95% quantile (shaded region) of estimate presented on logarithmic scale to be consistent with the rest of the manuscript. Values of permutation entropy close to 0 indicate low year-to-year variation in cases while values closer to 1 indicate high interannual variation. Minimum extreme winter temperatures correspond to Plant Hardiness Zones 3a-10b.


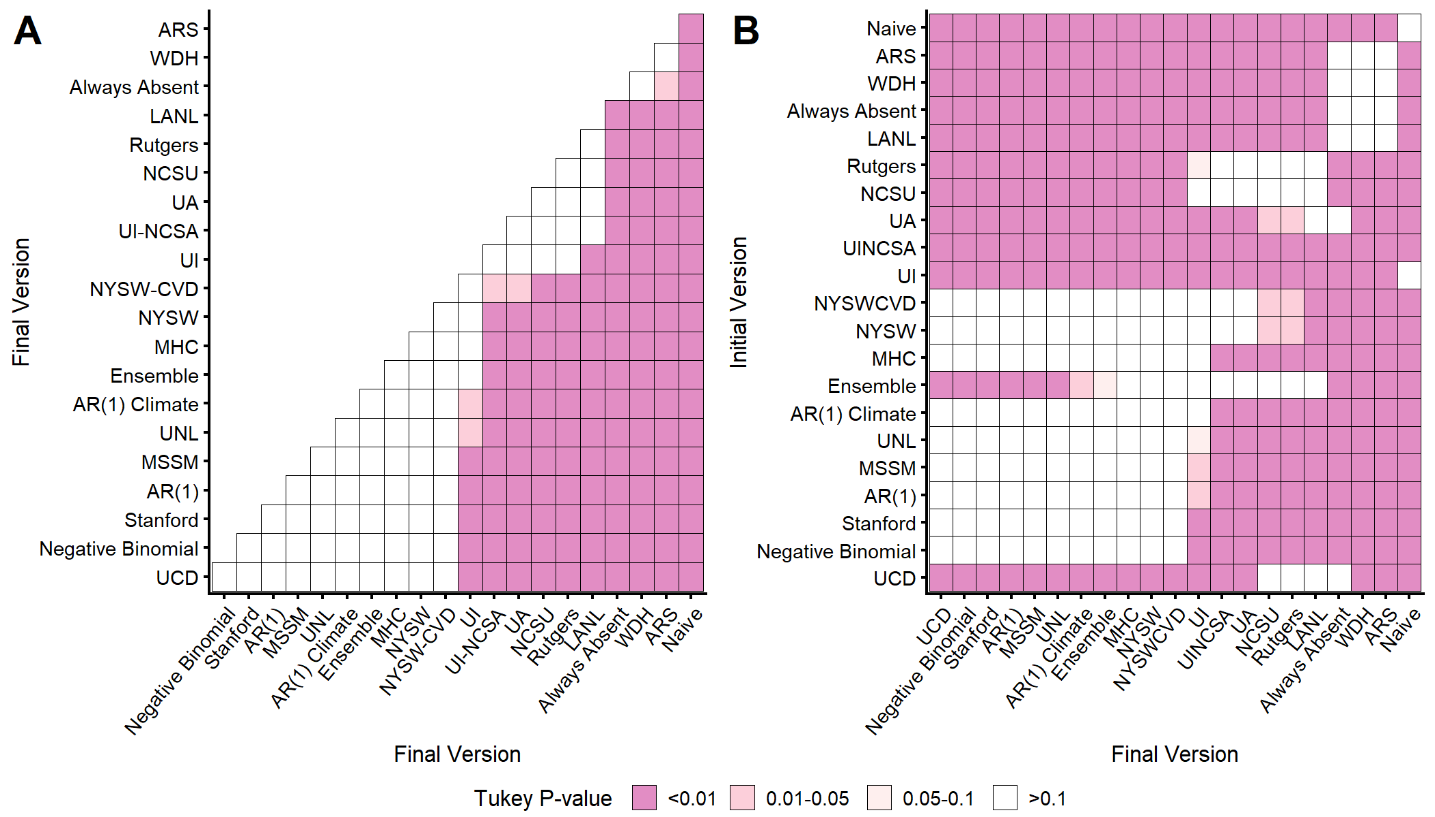


**Fig S4**. **Significance of difference in mean logarithmic score between forecasts.** Difference between A) final versions of teams and comparison models and B) the initial and final versions of forecasts. Significance calculated using Tukey post-hoc test. Forecasts ordered (left to right and bottom to top) by decreasing mean logarithmic score of the final version so that the best scoring model is at the lower left in each panel.


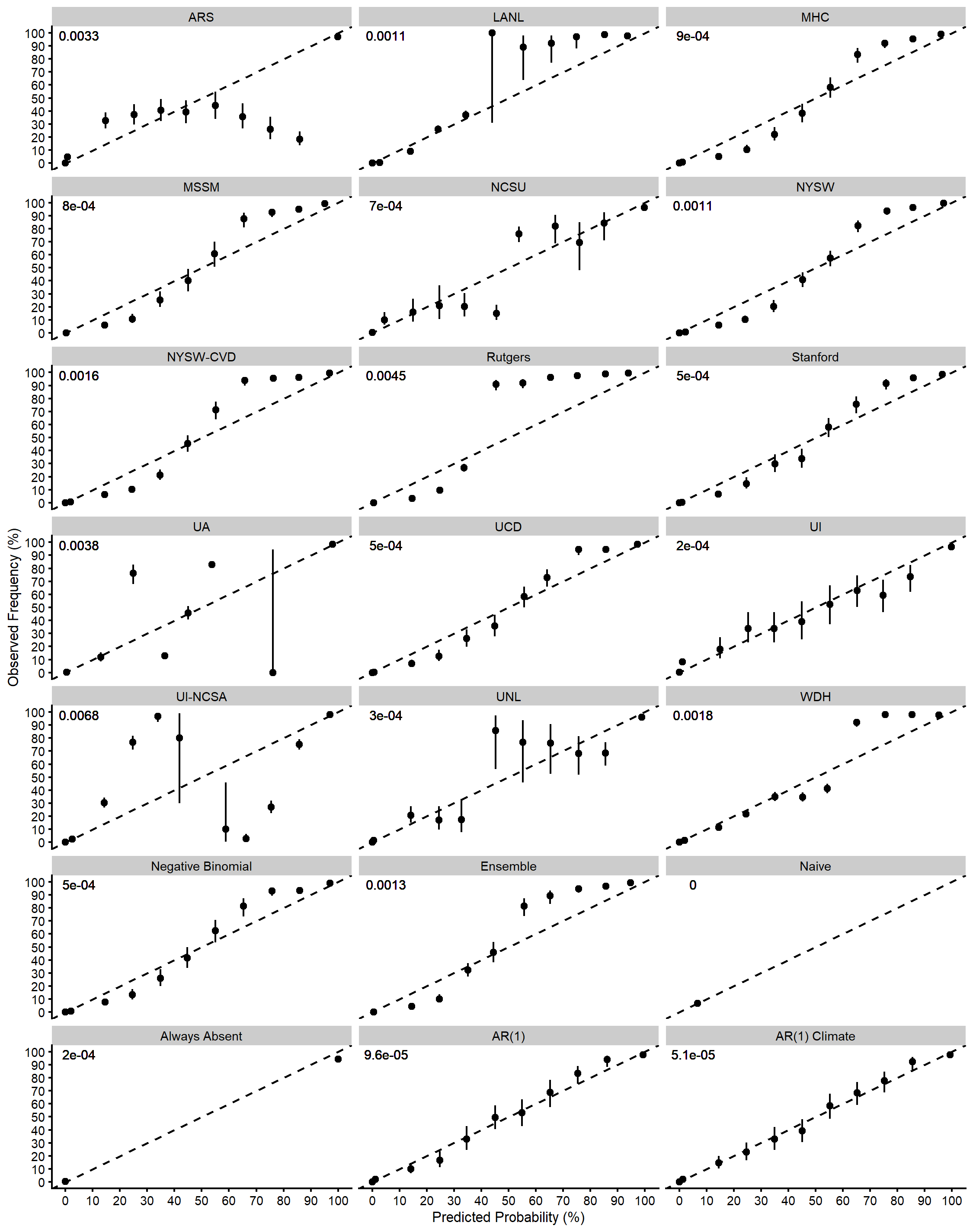


**Fig S5.** **Calibration of forecasts by teams and comparison models.** Each point indicates the average predicted probability within a given bin (e.g., between 0.2 and 0.3; x-axis) and the frequency of the observed outcome for those predictions (y-axis). The vertical line is the confidence interval for that observed frequency, where a large interval indicates fewer predictions in that bin. The dashed diagonal line indicates perfect calibration and the number in the top left is the calibration metric for that forecast, indicating distance from the line (smaller values better). Calibration calculated as the mean weighted squared difference of predicted probabilities vs. observed frequency of events.


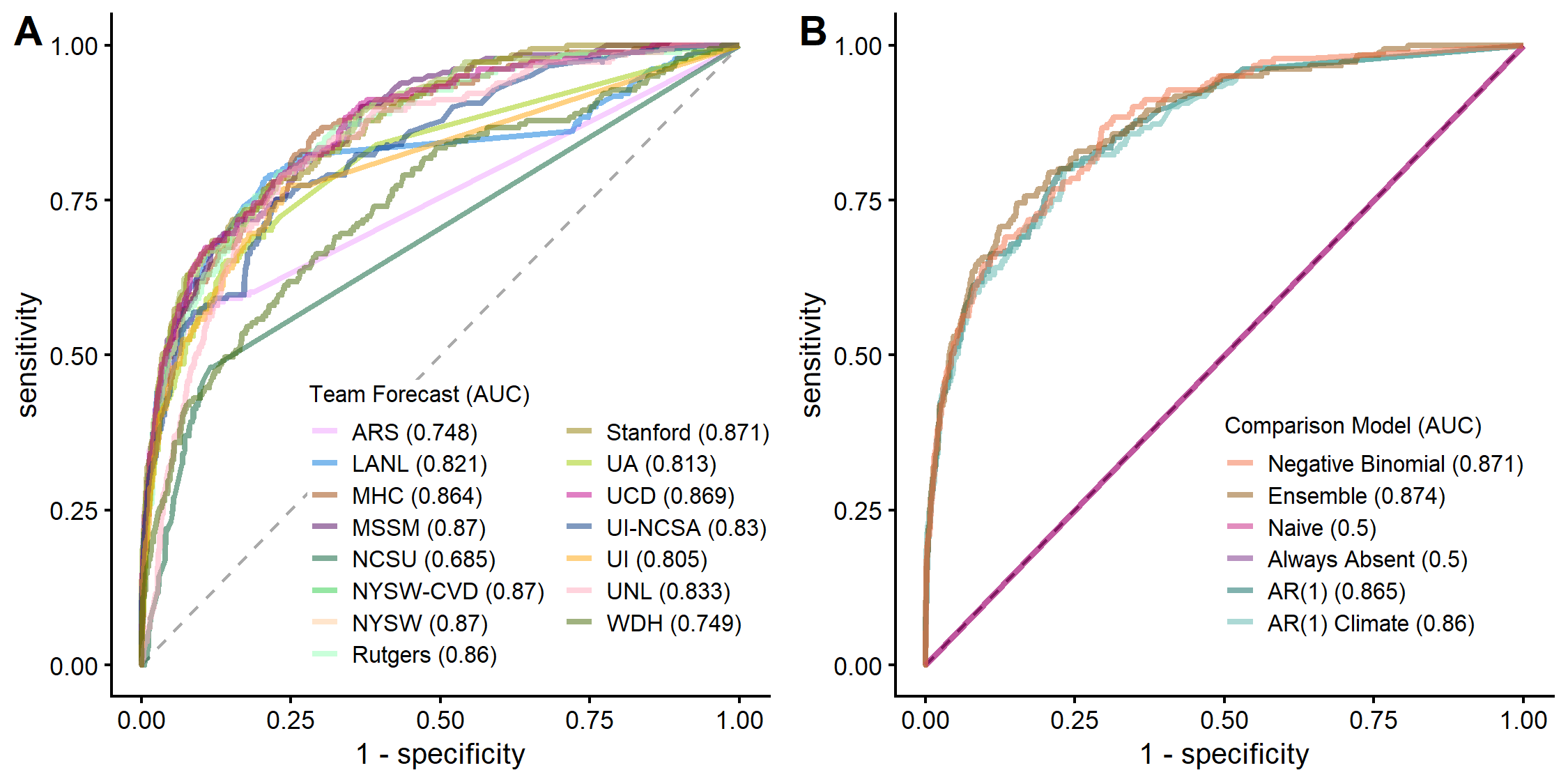


**Fig S6. Receiver Operator Characteristic (ROC) curves for forecasts by A) teams and B) comparison models.** All forecasts are the last submitted version for each. Area under the curve (AUC), a measure of discriminatory power of each forecast to predict counties with ≥ 1 reported WNV neuroinvasive disease case, indicated in legend. Diagonal dashed line in each panel represents random chance (AUC of 0.5).

**Table S1.** **Model characteristics and classes of covariates included in each team’s model.**

|  | Model Characteristics | | | Covariate Classes | | | | |
| --- | --- | --- | --- | --- | --- | --- | --- | --- |
| Team | **Type** | **Mech^+^** | **Bayesian** | **Climate data** | **Mosquito data*** | **Human demographics** | **Avian or equine cases** | **Land use** |
| ARS | Hur | No | **Yes** | **Yes** | **Yes** | **Yes** | **Yes** | No |
| LANL | Inc | No | No | No | No | No | No | No |
| MHC | Reg | No | **Yes** | No | No | **Yes** | No | No |
| MSSM | Inc | No | No | No | No | No | No | No |
| NCSU | DE | **Yes** | **Yes** | **Yes** | No | **Yes** | No | No |
| NYSW | ML | No | No | **Yes** | **Yes** | **Yes** | No | No |
| NYSW-CVD | ML | No | No | **Yes** | **Yes** | **Yes** | No | No |
| Rutgers | Reg | No | **Yes** | **Yes** | No | **Yes** | No | **Yes** |
| Stanford | ML | **Yes** | No | **Yes** | **Yes** | **Yes** | **Yes** | **Yes** |
| UA | ML | No | No | **Yes** | No | **Yes^** | No | No |
| UCD | Hur | No | **Yes** | **Yes** | No | **Yes** | No | **Yes** |
| UI | Reg# | No | No | **Yes#** | No | **Yes#** | No | **Yes#** |
| UI-NCSA | ML | No | No | **Yes** | No | No | No | **Yes** |
| UNL | Reg | No | No | **Yes** | No | No | No | No |
| WDH | Reg | No | **Yes** | No | No | **Yes** | No | No |

Teams arranged alphabetically by team name reflecting the affiliation of the team lead.

*^+^ Yes if model included any mechanistic pieces*

*^*^ Utilized geographic mosquito ranges/distributions or reported counts to ArboNet*

*^ UA model for April submission did not include demographics covariates while the model for July submission did include demographics covariates.*

*# UI April submission was a naive model with equal probability assigned to each bin (no covariates) while the following submission utilized zero-inflated Poisson regression with climate, demographics, and land use covariates (see Forecast Methods for more details).*

*DE: system of difference equations; Inc: historical incidence distribution; Hur: spatio-temporal hurdle model fitted using integrated nested Laplace approximation; ML: machine learning method like random forest or neural network; Reg: maximum likelihood generalized linear model or generalized additive model regression*

**Table S2**. **Reported West Nile virus neuroinvasive and non-neuroinvasive disease cases (2000-2020).**

| **Year** | **# Neuroinvasive cases** | **# Non-neuroinvasive cases** | **Ratio^** |
| --- | --- | --- | --- |
| 2000 | 19 | 2 | 9.50 |
| 2001 | 64 | 2 | 32.00 |
| 2002 | 2946 | 1,210 | 2.43 |
| 2003 | 2866 | 6,996 | 0.41 |
| 2004 | 1148 | 1,391 | 0.83 |
| 2005 | 1309 | 1,691 | 0.77 |
| 2006 | 1495 | 2,774 | 0.54 |
| 2007 | 1227 | 2,403 | 0.51 |
| 2008 | 689 | 667 | 1.03 |
| 2009 | 386 | 334 | 1.16 |
| 2010 | 629 | 392 | 1.60 |
| 2011 | 486 | 226 | 2.15 |
| 2012 | 2873 | 2,801 | 1.03 |
| 2013 | 1267 | 1,202 | 1.05 |
| 2014 | 1347 | 858 | 1.57 |
| 2015 | 1455 | 720 | 2.02 |
| 2016 | 1309 | 840 | 1.56 |
| 2017 | 1425 | 672 | 2.12 |
| 2018 | 1658 | 989 | 1.68 |
| 2019 | 633 | 338 | 1.87 |
| 2020 | 559 | 172 | 3.25 |

Cases reported in the contiguous states and Washington DC [12].

*^ Ratio of reported neuroinvasive to non-neuroinvasive cases.*

**Table S3**. **Mean logarithmic score for each team’s submitted forecast and six comparison models**.

|  | **Submission month** | | | |
| --- | --- | --- | --- | --- |
| **Team** | **April** | **May** | **June** | **July** |
| UCD | -0.509 | -0.208 | – | – |
| Negative Binomial | -0.218 | -0.215 | – | – |
| Stanford | -0.224 | – | – | – |
| AR(1)^+^ | -0.227 | – | – | – |
| MSSM | -0.229 | – | – | – |
| UNL | – | -0.241 | – | – |
| AR(1) Climate^#^ | -0.244 | – | – | – |
| Ensemble | -0.357 | -0.340 | -0.339 | -0.253 |
| MHC | -0.269 | – | – | – |
| NYSW | -0.301 | -0.285 | -0.274 | -0.272 |
| NYSW-CVD | -0.301 | -0.285 | -0.293 | -0.289 |
| UI | -2.708 | – | – | -0.340 |
| UI-NCSA | -0.971 | – | – | -0.390 |
| UA | -0.523 | – | – | -0.392 |
| NCSU | -0.412 | – | – | – |
| Rutgers | -0.443 | -0.415 | – | -0.413 |
| LANL | -0.679 | – | -0.449 | – |
| Always Absent | -0.582 | – | – | – |
| WDH | -0.643 | – | – | – |
| ARS | -0.679 | -0.679 | – | -0.679 |
| Naive | -2.708 | – | – | – |

Teams in descending order based on mean logarithmic score for last submitted forecast. Comparison models: negative binomial, ensemble, naïve, always absent, AR(1), and AR(1) Climate.

*– No new forecast submitted for this submission deadline.*

*^+^First-order auto-regressive model.*

*^#^ First-order auto-regressive model with climate covariate (minimum Dec-Feb (“winter”) temperature).*

**Table S4.** **Regression coefficients from Bayesian generalized linear model for modeling approaches associated with variation in skill.**

| coefficient^+^ | 50% quantile | 2.5%, 97.5% quantiles | ELPD (SE) | RMSE | MAE |
| --- | --- | --- | --- | --- | --- |
| climate | 0.187 | 0.174, 0.226 | 75114.7 (902.1) | 1.049 | 0.559 |
| land use | -0.100 | -0.124, -0.031 |  |  |  |
| mosquito | -0.142 | -0.114, -0.048 |  |  |  |
| demographics | 0.335 | 0.326, 0.361 |  |  |  |
| monthMJJ^ | 0.366 | 0.358, 0.390 |  |  |  |

Forecasts from teams and negative binomial comparison model used in analysis. Coefficient median (95% confidence interval) presented on logarithmic score.

*^+^ Coefficient for inclusion of each covariate class (climate, land use, mosquito data, and human demographics). Mosquito data included geographic mosquito ranges/distributions or reported counts to ArboNet.*

*^ Forecast submitted in May, June, or July (as compared to April) to capture updates to model structure or inclusion of new data.*

*ELPD: leave-one-out expected log pointwise predictive density; SE: standard error; RMSE: root mean squared error; MAE: mean absolute error*
